# Fungal Pathogens Associated with Crown and Root Rot of Wheat in Central, Eastern, and Southeastern Kazakhstan

**DOI:** 10.3390/jof8050417

**Published:** 2022-04-19

**Authors:** Tuğba Bozoğlu, Sibel Derviş, Mustafa Imren, Mohammed Amer, Fatih Özdemir, Timothy C. Paulitz, Alexey Morgounov, Abdelfattah A. Dababat, Göksel Özer

**Affiliations:** 1 Department of Plant Protection, Faculty of Agriculture, Bolu Abant Izzet Baysal University, Bolu 14030, Turkey; bzglu.tugba24@gmail.com (T.B.); m.imren37@gmail.com (M.I.); 2 Department of Plant and Animal Production, Vocational School of Kızıltepe, Mardin Artuklu University, Mardin 47000, Turkey; 3 Department of Mechanical Engineering, National Yang Ming Chiao Tung University, Hsinchu 30010, Taiwan; mohammedamer.me03g@g2.nctu.edu.tw; 4 Bahri Dagdas International Agricultural Research Institute, Konya 42050, Turkey; ozdemirfatih@tarimorman.gov.tr; 5 Wheat Health, Genetics and Quality Research Unit, United States Department of Agriculture, Agricultural Research Service, Washington State University, Pullman, WA 99164, USA; timothy.paulitz@usda.gov; 6 Food and Agriculture Organisation (FAO), Riyadh 11421, Saudi Arabia; alexey.morgounov@gmail.com; 7 International Maize and Wheat Improvement Centre (CIMMYT), P.O. Box 39, Emek, Ankara 06170, Turkey

**Keywords:** *Triticum* spp., wheat diseases, Fusarium crown rot, common root rot, soilborne diseases, pathogenicity

## Abstract

Kazakhstan is the fourteenth largest wheat producer in the world. Despite this fact, there has not been a comprehensive survey of wheat root and crown rot. A quantitative survey was conducted for the purpose of establishing the distribution of fungi associated with root and crown rot on wheat (*Triticum* spp.). During the 2019 growing season, samples were taken from the affected plants’ roots and stem bases. A total of 1221 fungal isolates were acquired from 65 sites across the central (Karagandy region), eastern (East Kazakhstan region), and southeastern (Almaty region) parts of the country and identified using morphological and molecular tools. The internal transcribed spacer (ITS), translation elongation factor 1-alpha (EF1-α), and glyceraldehyde-3-phosphate dehydrogenase (GAPDH) sequences were successfully used to identify the species of fungal isolates. It was found that *Bipolaris* *sorokiniana* (44.80%) and *Fusarium* *acuminatum* (20.39%) were the most predominant fungal species isolated, which were present in 86.15 and 66.15% of the fields surveyed, respectively, followed by *F*. *equiseti* (10.16%), *Curvularia* *spicifera* (7.62%), *F*. *culmorum* (4.75%), *F*. *oxysporum* (4.10%), *F*. *redolens* (2.38%), *Rhizoctonia* *solani* AG2-1 (1.06%), *Nigrospora* *oryzae* (0.98%), *C*. *inaequalis* (0.90%), *F*. *pseudograminearum* (0.74%), *F*. *flocciferum* (0.74%), *Macrophomina* *phaseolina* (0.66%), *F*. cf. *incarnatum* (0.33%), *Fusarium* sp. (0.25%), and *F*. *torulosum* (0.16%). A total of 74 isolates representing 16 species were tested via inoculation tests on the susceptible *Triticum aestivum* cv. Seri 82 and the results revealed that *F*. *culmorum* and *F*. *pseudograminearum*, *B*. *sorokiniana*, *Fusarium* sp., *R*. *solani*, *F*. *redolens*, *C*. *spicifera*, *C*. *inaequalis*, and *N*. *oryzae* were virulent, whereas others were non-pathogenic. The findings of this investigation demonstrate the presence of a diverse spectrum of pathogenic fungal species relevant to wheat crown and root rot in Kazakhstan. To the best of our knowledge, this is the first report of *F. pseudograminearum*, *Fusarium* sp., *C*. *spicifera*, and *C*. *inaequalis* as pathogens on wheat in Kazakhstan.

## 1. Introduction

Wheat (*Triticum* spp.) is a primary source of calories and protein [1], grown on 219 million ha and yielding 760.9 million tons [2]. Kazakhstan ranks fourteenth in wheat production, with 14.3 million tons produced on 12.1 million ha [2]. However, due to biotic and abiotic stressors, Kazakhstan’s wheat yield (1182.5 ton/ha) falls far short of the global average (3474.4 ton/ha).

In terms of biotic stress factors, the majority of reports in the country focused on airborne fungal foliar diseases, which were a recurring concern. Wheat stem rust (black rust) epidemics caused by biotrophic fungus *Puccinia graminis* f. sp. *tritici* were observed in the northern and eastern regions of Kazakhstan from 2015 to 2018, affecting approximately one million ha of wheat and resulting in a significant drop in yield, as well as inferior grain quality [3,4]. The pathogen’s race composition was also explored in the Akmola and Kostanay regions, as well as in East Kazakhstan [4]. Furthermore, the development of *Puccinia triticina* (syn: *P*. *recondita* f. sp. *tritici*), another common representative of the *Puccinia* genus, on spring wheat in Northern Kazakhstan’s Akmola region during 2000–2001 resulted in 50–100% leaf rust (brown rust) severity on commercial cultivars [5]. Afterward, 46 pathotypes of *P*. *striiformis* f. sp. *tritici*, the causal agent of stripe or yellow rust, were determined in a population collected from Kazakhstan’s southern and southeastern regions [6]. The pathogen *Pyrenophora tritici-repentis*, which causes tan spot and is one of the wheat yield-limiting diseases, has also been reported to be rapidly spreading in Kazakhstan [7]. Furthermore, in 126 samples collected from North Kazakhstan, the prevalence of *P*. *tritici-repentis* isolates was 43% [8]. Spot blotch caused by *Cochliobolus sativus* (anamorph *Bipolaris sorokiniana*) and glume blotch (or *Stagonospora nodorum* blotch) caused by *Parastagonospora nodorum* (syn. *Septoria nodorum*, anamorph *Stagonospora nodorum*) were also reported to be prevalent diseases causing significant yield reductions in wheat in Kazakhstan [9,10], but no scientific report confirming these statements could be obtained. The majority of the aforementioned fungal diseases, stem, leaf, and stripe rusts, and tan spot, are the most serious threats to wheat production worldwide, along with Fusarium head blight caused by 17 species of *Fusarium*, the most common of which is *F. graminearum*, powdery mildew caused by *Blumeria graminis* f. sp. *tritici*, *Septoria tritici* blotch caused by *Zymoseptoria tritici* (syn. anamorph *Septoria tritici*; teleomorph *Mycosphaerella graminicola*), and the wheat blast caused by *Magnaporthe oryzae* (anamorph *Pyricularia oryzae*) [11]. Meanwhile, and based on our knowledge, wheat root- and crown root-associated fungi have never been explored within the country.

A variety of root and stem base rot complex diseases frequently hinder wheat yields, stands, and grain quality in wheat-growing countries [12,13,14]. This complex can be caused by a variety of infections that cause damping-off, blight, necrosis, and dry rotting of the root, crown, sub-crown, and lower stem tissues, along with wilting and stunting of seedlings and mature wheat plants [15]. Infections of these pathogens in the root and stem base compartments of wheat have a significant impact on the number of tillers as well as the number and size of kernels, and severe infections in the root and crown of seedlings can be fatal [14]. Various ubiquitous or soilborne fungal pathogens occurring on the same plant and in the same field are associated with this complex, but common root rot caused by *B. sorokiniana* and Fusarium crown rot caused by multiple *Fusarium* species, which create a challenge for disease diagnosis and detection and require a combination of conventional and molecular-based methods for diagnosis, are the most common and have global significance. *Fusarium pseudograminearum*, *F*. *culmorum*, and *F*. *graminearum* are the most common *Fusarium* species that cause crown and root rot in wheat [16,17,18,19]. *Fusarium avenaceum*, *F*. *acuminatum*, *F*. *equiseti*, *F*. *crookwellense*, *F*. *poae*, *F*. *chlamydosporum*, *F*. *algeriense*, and dozens of other *Fusarium* species, as well as *Microdochium nivale* (formerly known as *Fusarium nivale*), *M*. *majus*, *Gaeumannomyces graminis*, *Rhizoctonia* spp., and the oomycete *Pythium* spp., are minor contributors to wheat yield losses in this complex and are sporadically problematic in specific environments and agroecological zones [15,16,19,20,21,22,23]. These fungi have complex population dynamics and interact with one another in crop residues, soil, and seed to survive between wheat crops. Multiple fungal species frequently co-occur at the same time and may interact in synergistic or competing ways, influencing their development and disease-causing capabilities [24]. Furthermore, the composition of these pathogens and the severity and incidence of the disease that they cause is influenced by various factors, such as the pathogen populations in the soil, wheat genotype, time of infection, season, soil type, temperature, moisture, nutrition, geographical region, cropping sequence, suppressive capacity of the soil microbiome, and tillage and other cultural practices [14,19,25,26,27,28,29]. Additionally, some species such as *F*. *graminearum*, *F*. *avenaceum*, *F*. *culmorum*, *F*. *poae*, *M*. *nivale*, and *M*. *majus*, which can be seed-borne, soilborne, or residue-borne, cause a variety of diseases in wheat during its development, including seedling blight, root rot, foot/crown rot, and head blight [30,31]. Similarly, *B*. *sorokiniana* causes a number of diseases, including common root rot, spot blotch, and black point on wheat roots, leaves, and kernels, in that order [29,32]. The pathogen also can attack other wheat organs, such as the crown and stems. This means that relevant strategies for such diseases should not only focus on limiting the presence of fungi in aerial plant parts but also pathogens on the crown and root tissues, as well as inoculum presence in soil. For outbreaks of wheat crown and root rot, the interplay of fungal development with the geographical and temporal variability of all of these parameters is critical. As a result of the disease’s complexity, cereal producers and scientists have struggled to manage it for decades [33]. To combat the disease, a thorough understanding of pathogen biology and populations, as well as the epidemiological factors that contribute to disease exacerbation, is required. The first step in this process is to collect data on the distribution and prevalence of wheat pathogens associated with this phenomenon, especially since these data could be beneficial in predicting future changes in disease distribution patterns.

In Kazakhstan, there is a lack of knowledge regarding the distribution and frequency of diseases in wheat underground and crown tissues. Although wheat crown and common root rot have become a serious problem in some parts of Kazakhstan in recent years, only two reports on root and crown rot from Kazakhstan have been published to date, and the presence of *F*. *redolens* [34] and *R*. *solani* AG2-1 [35], the causative agents of crown and root rot, was confirmed in the six and four wheat fields surveyed, respectively. Additionally, 500 wheat seeds collected from various localities in the country’s Astana Province were analyzed for mycoflora using an agar plate method, and, in a Petri dish assay, *Nigrospora oryzae* was identified as a seed pathogen of wheat that causes lesions on the hypocotyls of infected seeds [36].

In this study, a detailed survey study was conducted to assess the distribution of pathogen populations associated with crown and root rot across commercial wheat fields in the central (Karagandy region), eastern (East Kazakhstan region), and southeastern (Almaty region) parts of the country in order to generate information and understand disease dynamics, as well as to test the pathogenicity of the obtained species on a susceptible wheat cultivar.

## 2. Materials and Methods

### 2.1. Sample Collection

**Figure 1:**
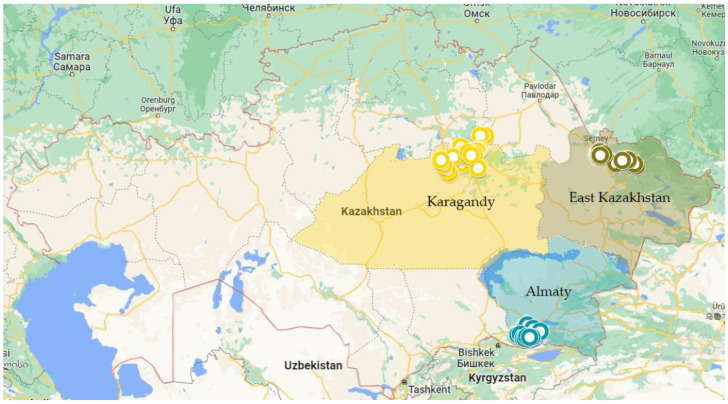
Map of the Republic of Kazakhstan showing the different agro-geographical regions and sampling sites during July 2019.

Plant samples with typical crown and root rot symptoms were collected from 65 wheat fields (26 from Karagandy, 18 from East Kazakhstan, and 21 from Almaty regions) in the summer of 2019 at the crop’s maturity stage and harvesting time (Figure 1). The surveyed fields were arbitrarily chosen, with a distance of 5–15 km apart. From an area of 5-ha, 20–30 symptomatic wheat plants were randomly sampled in a zig-zag pattern, with each plant being 20 m apart and placed in paper bags. Samples were brought to the laboratory and kept at 4 °C until fungal isolation.

### 2.2. Isolation and Maintenance of Cultures

Collected plants were cut approximately 25 cm above the base of the stem. Before examining the stems for lesions, the crown, root, and stem base tissues were washed thoroughly under tap water for 15 min to remove soil particles. Small portions of necrotic and discolored crown, sub-crown, and root tissues of sampled plants were superficially disinfested for 1 min with 1% sodium hypochlorite solution, rinsed with sterile water, and blotted dry. The dried sections were chopped into 1 cm lengths and placed on 1/5 strength potato dextrose agar (PDA; diced potato tubers (40 g) were cooked in water for 15 min, the strained broth was brought to 1 L, and dextrose (4 g) and agar (15 g) were added and then autoclaved) fortified with streptomycin (0.1 g/L) and chloramphenicol (0.05 g/L) to inhibit bacterial contamination. Fungal colonies isolated from the sections were sub-cultured on new full-strength PDA (200 g diced potato tubers, 20 g dextrose, and 15 g agar) plates and purified using the hyphal tip or the single spore isolation method after five days of incubation in the dark at 23 °C. *Fusarium*-like colonies were also transferred to Spezieller Nährstoffarmer Agar (SNA) medium (1 g KH_2_PO_4_, 1 g KNO_3_, 0.5 g MgSO_4_·7H_2_O, 0.5 g KCl, 0.2 dextrose, 0.2 sucrose, 20 g agar, distilled water to 1 L) and incubated at 23 °C for 10 days to enhance conidia and chlamydospore production [37]. Fungi were routinely maintained on PDA in the dark at 23 °C. All isolates were kept at 4 °C on PDA slants during studies and stored at −80 °C in vials containing 15% glycerol for long-term storage.

### 2.3. DNA Extraction

Genomic DNA was isolated using a DNeasy Blood and Tissue Kit (Qiagen, Hilden, Germany) according to the manufacturer’s instructions. Fungal mycelia with spores were collected by gently scraping the surfaces of 7-day-old PDA cultures incubated at 23 °C and were ground to a powder in liquid nitrogen. Template DNA was extracted from 50–100 mg of fungal powder and dissolved in 1× TE buffer (1 mM EDTA, 10 mM Tris-HCl, pH 8.0). DNA concentration was estimated spectrophotometrically by the A260/A280 ratio using a DS-11 FX+ nano spectrophotometer (Denovix Inc., Wilmington, DE, USA). Before further analyses, the DNA extract was diluted to 10 ng/μL and stored at −20 °C.

### 2.4. Species Identification and Phylogenetic Analysis

Morphological identifications of *Fusarium* spp. isolates were primarily performed based on microscopic characteristics, colony appearance, and pigmentation, as described by Leslie and Summerell [37], whereas dematiaceous fungal isolates were identified using Ellis [38] and Sivanesan’s [39] key. The remaining species, *Nigrospora* sp. and *Macrophomina* sp., were identified using Hudson’s [40] and Holliday and Punithalingam’s [41] descriptions, respectively.

The translation elongation factor 1- alpha (EF1-α) gene for *Fusarium* spp. and the internal transcribed spacer (ITS) region of ribosomal DNA for other isolates were amplified with EF1/EF2 [42] and ITS1/ITS4 [43] primer sets, respectively. The glyceraldehyde-3-phosphate dehydrogenase (GAPDH) locus was also amplified for *Cochliobolus* anamorphs using gpd1/gpd2 primers described by Berbee et al. [44]. The PCR mixture contained 1× PCR reaction buffer, 1.5-unit Ampliqon TEMPase Hot Start DNA polymerase (Berntsen, Rdovre, Denmark), 0.4 µM of each primer, 0.2 mM of each dNTP, 10 ng template DNA, and sterile milli-Q water up to 50 μL. The PCR amplification was performed with an initial denaturation at 95 °C for 15 min, followed by 95 °C for 45 s, annealing at 54 °C for 45 s, and extension at 72 °C for 60 s for 35 cycles, and a final extension at 72 °C for 10 min, conducted in a T100 thermal cycler (Bio-Rad Laboratories, Hercules, CA, USA). The PCR products were bi-directionally sequenced by Macrogen Inc. (Seoul, Korea) with the same primers.

The DNA sequences were edited, and consensus sequences were computed manually using Mega X: Molecular Evolutionary Genetics Analysis across computing platforms [45]. All sequences were compared against the GenBank database, National Center for Biotechnological Information, using the BLASTn algorithm, and representative isolates for each species were deposited in GenBank. *Fusarium* isolates and other isolates were phylogenetically analyzed separately based on the EF1-α and ITS sequences, respectively. The obtained isolates from this study with additional reference sequences and 2 outgroups retrieved from the GenBank database were aligned in the MAFFT v.7 online interface ([46], http://mafft.cbrc.jp/alignment/server/) using default settings, and manually edited with MEGA X. A maximum likelihood (ML) tree of the EF1-α and ITS data set was separately inferred using the command-line version of IQ-TREE 1.6.7 [47] with ultrafast bootstrapping implemented with 1000 replicates. The phylogenetic analysis was also performed using the Bayesian method using MrBayes 3.2.7 [48] run on the XSEDE with 1.00 posterior possibilities. Analyses were run on the CIPRES Science Gateway V 3.3. ([49], https://www.phylo.org/). The resulting trees were analyzed and edited in FigTree v1.4.2 software. The EF1-α sequence of *Fusarium ventricosum* strain CBS 748.79 (KM231924) and the ITS sequence of *Phytophthora infestans* strain CBS 120920 (MF680417) were added as outgroups to facilitate the production of consensus trees.

### 2.5. Isolation Frequency and Incidence of Fungal Species in the Fields

Isolation frequency and field incidence of fungal species were estimated after species identification of each isolate. Isolation frequency was determined by dividing the number of fungal isolates per species by the total number of isolates obtained and expressing the value as a percentage. The incidence of individual species in the fields was calculated by dividing the number of locations from which fungal species were retrieved by the total number of fields surveyed and expressing the value as a percentage.

### 2.6. Pathogenicity Tests

Following species identification, 74 isolates representing 16 species (two to five isolates of each species) were chosen from various locations to ensure geographic representation and pathogenicity testing on wheat seedlings under growth room conditions of 12/12 h light/dark regime and at 23 °C. Common bread wheat (*Triticum aestivum* L.) cv. Seri 82 seeds were sterilized for 2 min in 1% NaOCl and then placed in plates containing a piece of sterile filter paper saturated with water to enhance germination. Plastic pots (15 cm diameter and 17 cm height) were filled with a potting mixture of peat (KTS 1, Klasmann-Deilmann, Germany), sterile vermiculite, and sterile soil (1:1:1, *v/v/v*). Five identical seedlings were placed on the soil surface of each pot. For inoculation of *Fusarium* spp. isolates, mycelial plugs (9 mm diameter) were cut from the margin of a growing PDA culture of representative isolates and placed onto the mixture substrate in the pots; then, the seedlings and the mycelial plugs were covered with the same potting mixture [23]. A sterile agar plug was used as a control treatment. Each seedling was placed on a mycelial plug and covered with the mixture substrate. To test the pathogenicity of dematiaceous fungal isolates (*B*. *sorokiniana*, *C*. *spicifera*, and *C*. *inaequalis*) and *N*. *oryzae*, a conidial suspension of each isolate was injected into the potting mixture used to cover the seedlings at a density of 250 conidia per gram [50]. The pathogenicity of *R*. *solani* AG2-1 and *M*. *phaseolina* isolates was assessed using the colonized wheat kernels method, which was developed from Demirci [51], by placing 10 colonized wheat kernels in contact with wheat seedlings before being covered with the potting mixture. The control treatment received sterile wheat kernels. Five seeds were planted in each pot, and three replicated pots were employed for each isolate. All experiments were conducted twice. Plants were uprooted, washed, and examined for discoloration or lesions on the crown, sub-crown internode, and root tissues six weeks after incubation at a 12 h photoperiod at 23 °C. The index system with a 1–5 scale of Nicol et al. [52] (modified from Wildermuth and McNamara [53]) was used to assess the disease symptoms, which were based on the percentage of typical browning/rot at the crown and base of the stem (1: 1–9% Resistant, 2: 10–29% Medium durable, 3: 30–69% Medium sensitive, 4: 70–89% Sensitive, 5: 90–100% Very sensitive). The experiments were conducted twice. The mean disease ratings of each isolate in the pathogenicity assay were determined on 15 replicated seedlings (five seedlings per pot). Mean scores of 1–2 were considered non-pathogenic or mildly virulent if there were significant differences between treatment and control. Scores of 2–3 were considered moderately virulent, and scores higher than 3 were considered highly virulent.

Disease severity scores obtained from the pathogenicity tests were analyzed for significance by analysis of variance, followed by Fisher’s least significant difference test (LSD) at *p* = 0.05 with Statistical Analysis System (SAS Version 9.0; SAS Institute Inc.; Cary, NC, USA).

## 3. Results

A total of 1221 fungal isolates were isolated during the 2019 growing season from symptomatic wheat samples collected from 65 fields in Kazakhstan’s wheat-growing regions. Based on morphological and molecular tools, 547 isolates of *B*. *sorokiniana*, 537 of *Fusarium* spp., 93 of *C*. *spicifera*, 13 of *Rhizoctonia solani* AG2-1, 12 of *Nigrospora oryzae* (Berk. & Broome) Petch, 11 of *C*. *inaequalis*, and 8 of *Macrophomina phaseolina* (Tassi) Goid were identified (Table 1).

In the three regions surveyed, *B*. *sorokiniana*, the anamorph of *Cochliobolus sativus*, was the most frequently recovered species (43.31% to 47.49%) and had the highest field incidence (72.22% to 96.15%), which accounted for 44.80% of the isolates and 86.15% of the ‘fields’ surveyed.

**Figure 2:**
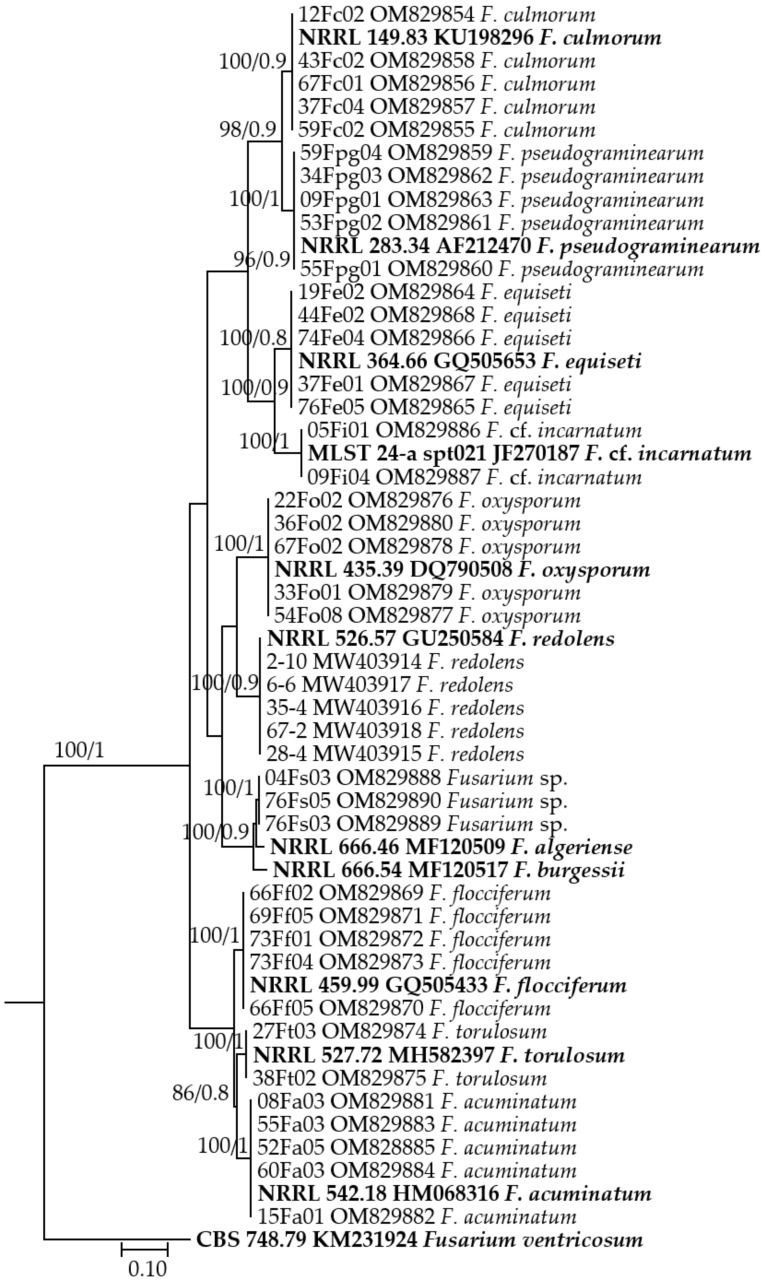
Phylogenetic tree based on maximum likelihood using IQ-TREE and Bayesian inference (BI) using MrBayes with EF1-α sequences of *Fusarium* isolates. At each node are the bootstrap values (left) and posterior probabilities (right). Sequences used as references and outgroups in the present study are presented in bold.

Isolates of *Fusarium* spp. comprised 43.98% of the total number of isolates and were classified into 10 *Fusarium* species by comparing them to the published descriptions and DNA sequencing. The EF1-α gene sequences of *Fusarium* spp. isolates ranged in length from 636 to 693 bp, with 100% identity matches to corresponding *Fusarium* species (except *Fusarium* sp.) in the GenBank database. Figure 2 shows the accession numbers for the representative sequences of *Fusarium* species deposited in the NCBI. *Fusarium acuminatum* and *F*. *equiseti* were the most frequent *Fusarium* species, accounting for 46.37% and 23.09% of *Fusarium* spp. isolates, respectively, followed by *F*. *culmorum* and *F*. *oxysporum*, which accounted for 10.80% and 9.31%, respectively. *Fusarium redolens* (Wollenw), *F*. *pseudograminearum*, *F*. *flocciferum* (Corda), *F*. cf. *incarnatum* (Subraman. & Rao), and *Fusarium* sp. were found less commonly, with frequencies of 5.40%, 1.68%, 1.68%, 0.74%, and 0.56%, respectively, whereas *F*. *torulosum* (Berk. & M.A. Curtis) Nirenberg was the least predominant (0.37%), and two isolates could be identified in one field from Karagandy and one field from East Kazakhstan. Except for *F*. *flocciferum* (4.62% incidence), *Fusarium* sp. (3.08% incidence), and *F*. cf. *incarnatum* (3.08% incidence), other *Fusarium* species could be found in each of the three surveyed regions, but the incidence of individual species varied among these regions (Table 2). Concerning individual species, *F*. *acuminatum* was found in 66.15% of the surveyed fields, *F*. *oxysporum* in 41.54%, *F*. *equiseti* in 35.38%, *F*. *culmorum* in 18.46%, *F*. *redolens* in 9.23%, and *F*. *pseudograminearum* in 7.69%.

Other anamorphs of *Cochliobolus* species, *C*. *spicifera,* and *C*. *inaequalis* were found in 7.62% and 0.90% of the isolates obtained and in 27.69% and 7.69% of the fields sampled, respectively. *Nigrospora oryzae* and *M*. *phaseolina* were isolated from 9.23% and 3.08% of the examined fields, respectively. Various fungal species were found frequently (92.31%) to coexist in a single field. For example, 547 isolates of *B*. *sorokiniana* and 249 isolates of *F*. *acuminatum* were identified in 86.15% and 66.15% of the 65 fields surveyed, respectively. The isolation frequency and field incidence of retrieved species varied by region. For instance, in Karagandy, all *Cochliobolus* anamorphs and *F*. *acuminatum* were isolated more frequently and had a higher field incidence than in East Kazakhstan and Almaty. All other species, if present in all or two of the regions, had higher isolation frequency and field incidence ratios in East Kazakhstan and Almaty than in Karagandy.

**Figure 3:**
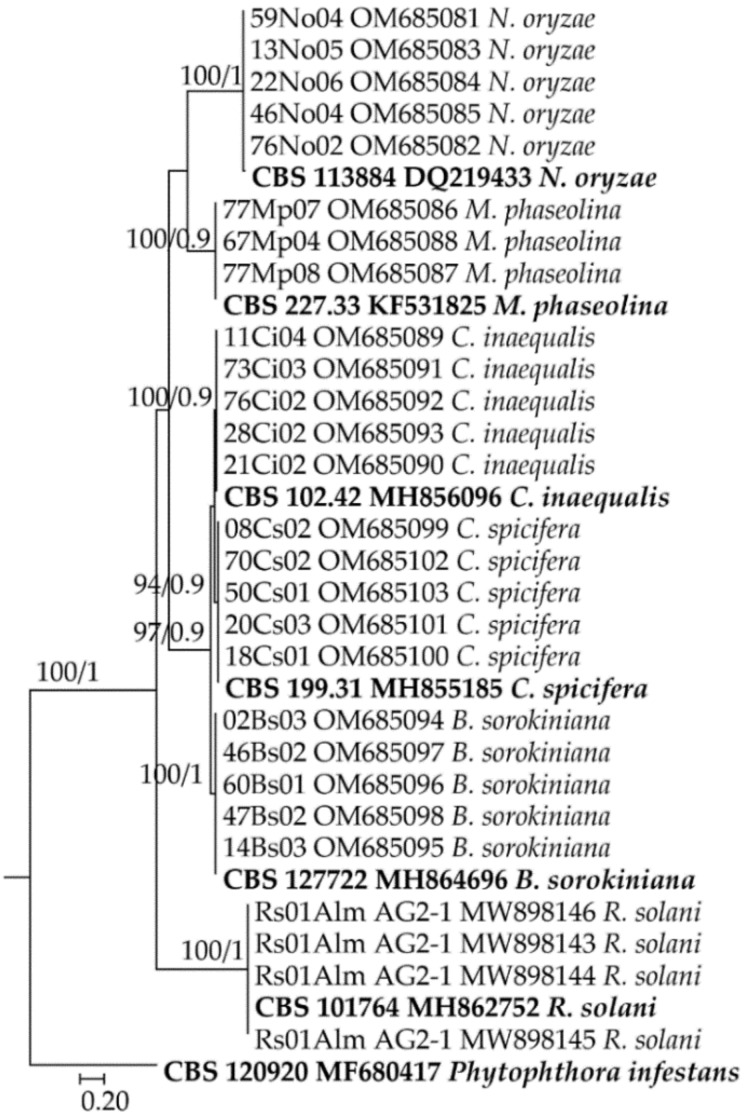
Phylogenetic tree based on maximum likelihood using IQ-TREE and Bayesian inference (BI) using MrBayes with ITS sequences of fungal isolates (except *Fusarium*). At each node are the bootstrap values (left) and posterior probabilities (right). Sequences used as references and outgroups in the present study are presented in bold.

BLASTn queries based on the ITS of isolates showed that the sequences of isolates were 99–100% identical to those of the corresponding species in the GenBank database. The sequences were deposited in GenBank under the accession numbers shown in Figure 3. Phylogenetic analyses based on the ITS sequences of the isolates studied and reference sequences of *B*. *sorokiniana*, *C*. *spicifera*, *C*. *inaequalis*, *M*. *phaseolina*, and *N*. *oryzae* available in GenBank indicated that the isolates belonging to the same species were clearly separated in the dendrogram (Figure 3). GAPDH sequences of anamorphs of *Cochliobolus* were also 100% identical to those of the corresponding species and deposited in GenBank under accession nos. OM937370-OM937384.

From eight field samples, thirteen morphologically identical *R*. *solani* isolates were identified. The anastomosis group determination of the isolates representing each field was conducted by the sequencing of the ITS region of rDNA. All isolates were determined as *R*. *solani* AG2-1. The ITS sequences of isolates were analyzed using the BLAST algorithm, the sequences were 100% identical to *R*. *solani* AG2-1 sequences in GenBank, and all isolates were clustered in the dendrogram with isolates belonging to the same AG.

The pathogenicity tests revealed that *F*. *pseudograminearum* and *F*. *culmorum* isolates proved to be the most and equally virulent pathogens (*p* < 0.05) and had a mean crown rot severity of 3.55 and 3.42, respectively (Table 3). Several isolates of both species caused substantial damage, with entire plants dying in some pots. *Bipolaris sorokiniana*, on the other hand, was moderately virulent on the crowns and roots, inducing necrosis or rot at similar levels as *Fusarium* sp. isolates. Likewise, *R*. *solani* AG2-1 and *F*. *redolens* isolates showed a mean crown rot severity of more than 2 and were moderately virulent but not as severe as the aforementioned species. However, isolates of *C*. *spicifera*, *C*. *inaequalis*, and *N*. *oryzae* were only mildly virulent, causing slight discoloration on a few crowns and seminal roots of inoculated plants, with a mean crown rot severity of 1.94, 1.60, and 1.56, respectively. *Fusarium acuminatum*, *F*. *oxysporum*, *F*. *flocciferum*, *F*. *torulosum*, *F*. cf. *incarnatum*, and *F*. *equiseti*, as well as *M*. *phaseolina*, were non-pathogenic on wheat seedlings, displaying no significant differences from control plants.

## 4. Discussion

Global food production continues to be challenged by an alarmingly growing population, a finite amount of arable land, and conflicts between the countries that are important in the world wheat trade. Wheat is severely threatened by rapid climate change, which affects the world’s abiotic and biotic components [54]. In an effort to address some of these challenges, field assessments of the crown and root rot pathogens on wheat, which harbors an array of pathogenic species, are reported on a regular basis in the world’s largest wheat-producing countries [25,55]. However, Kazakhstan, the world’s fourteenth largest wheat producer, does not have sufficient information about wheat root and crown rot diseases.

Various research and application purposes, as well as the establishment of effective control methods, are pursued in plant pathology and related disciplines using the identification, geographical distribution, and severity of fungal plant pathogens. As such, they represent a fundamental step for further development in this field. This study is the first to present a comprehensive survey of the pathogens associated with wheat crown and root rot in the Karagandy, East Kazakhstan, and Almaty regions of Kazakhstan. The disease was widely distributed throughout all surveyed areas, and anamorphs of *Cochliobolus* (*B*. *sorokiniana*, *C*. *spicifera*, and *C*. *inaequalis*), *N*. *oryzae*, 10 *Fusarium* species, *R*. *solani* AG2-1, and *M*. *phaseolina* were found on wheat stem bases and roots, based on the morphologic characteristics and ITS and EF1-α sequences of isolates. The four most common species in the majority of the sampling areas were *B*. *sorokiniana*, *F*. *acuminatum*, *F*. *oxysporum*, and *F*. *equiseti*, with field incidences of 86.15, 66.15, 41.54, and 35.38%, respectively. The order of isolation frequency for the first two species corresponded to the field incidence values, with *B*. *sorokiniana* and *F*. *acuminatum* having the highest importance. *Fusarium equiseti*, *C*. *spicifera*, and *F*. *culmorum*, on the other hand, had higher isolation rates than *F*. *oxysporum*. There have been numerous reports of root and crown rot-causing agents predominating in various geographic zones, including the United States [20,26], Canada [56], Chile [57], Australia [58], Turkey [17,59,60,61], China [19,62], and Azerbaijan [23].

Generally, the results provided here are in accordance with results provided by other studies conducted in wheat-growing regions around the world. *Bipolaris sorokiniana* was considered to be the major pathogen for this disease in Kazakhstan, with a high field incidence (86.15%) and isolation frequency from roots and crowns (44.80%). Despite the fact that it was the most common species in all of the regions studied, its field incidence varied depending on the agronomic zone. The incidence of *B*. *sorokiniana* increased by up to 96.15% when the plant was grown in cold soil, such as in Karagandy, contrary to the findings of Mathieson et al. [63] and Acharya et al. [64]. These *B*. *sorokiniana* ratios are quite similar to those reported from China, the world’s largest wheat producer, where it is described as a predominant pathogen of root and crown rot in wheat (field incidence: 82.7%, and isolation frequency from roots: 24%, and stems: 33.7%) [19], and the fungus is also recognized as one of the most devastating pathogens of wheat worldwide [12,29,64]. *Fusarium acuminatum*, *F*. *oxysporum*, and *F*. *equiseti* had high field incidence (35.38 to 66.15%) and isolation frequency (4.10 to 20.39%), which were similar to those previously reported for crown and root rot fungi in several countries. In dryland winter wheat-producing areas of Colorado and Wyoming, the most frequently associated fungi with common root rot were *B*. *sorokiniana* and *F*. *acuminatum*, which matched our findings [65]. However, when *B*. *sorokiniana* was inoculated alone, it caused significant disease, while, when plants were inoculated with both fungi, *F*. *acuminatum* significantly augmented the effects of *B*. *sorokiniana* [66]. Fernandez et al. [67] indicated that the most frequently detected fungi in Western Canada were *B*. *sorokiniana*, *F*. *equiseti*, *F*. *avenaceum*, *F*. *acuminatum*, and *F*. *oxysporum*. In Turkey, the most commonly isolated *Fusarium* species was *F*. *equiseti*, which accounted for 36% of all *Fusarium* species isolated [61]. Mississippi also had a high prevalence of *F*. *acuminatum* and *F*. *equiseti* [68]. Similar to our findings, a high frequency of *F*. *oxysporum* (27.4%) has been reported from Serbia [69]. Surprisingly, the relatively low field incidence (7.69% and 18.46%) and isolation frequency (0.74% and 4.75%) of *F*. *pseudograminearum* and *F*. *culmorum* differed from those previously reported for crown and root rot fungi [16,17,18,19,30,58,59,70]. In particular, the most prevalent species associated with this disease in Australia [71], North America [20], and Turkey [61] appears to be *F*. *pseudograminearum*. Similarly, in previous reports, *F*. *culmorum* has been identified as the main pathogen species of wheat [16,20]. Differences in climate and farming practices in the sampling locations could be responsible for these disparities among geographical populations. For instance, the distribution feature of the two most common *Fusarium* species, *F*. *acuminatum* and *F*. *oxysporum*, showed that *F*. *acuminatum* was found in 92.31% of the fields surveyed in Karagandy, whereas *F*. *oxysporum* appeared to be more widespread in East Kazakhstan and Almaty fields, with incidence rates exceeding 50%. Two other anamorphs of *Cochliobolus* (*C*. *spicifera* and *C*. *inaequalis*) were also more common in Karagandy, similar to *B*. *sorokiniana*. These findings imply that the distribution of individual species appears to be related to their adaptation to specific environments [16,72,73].

*Fusarium* sp. isolates are likely to be a novel crown rot pathogen of wheat within the *F*. *burgessii* complex, which includes *F*. *algeriense* and *F*. *burgessii*, according to phylogenetic analysis based on EF1-α sequences; however, more detailed morphological and molecular studies are needed to confirm this thesis.

The pathogenicity results of 74 isolates belonging to 17 fungal species confirmed previous findings of significant variation in visual discoloration ratings on wheat seedlings caused by different fungi. Four *Fusarium* species (*F.* *pseudograminearum*, *F*. *culmorum*, *Fusarium* sp., and *F*. *redolens*), *B*. *sorokiniana*, *R*. *solani* AG2-1, and all anamorphs of *Cochliobolus* examined, *B*. *sorokiniana*, *C. spicifera*, and *C*. *inaequalis*, and *N*. *oryzae*, were capable of causing crown, stem base, and root necrosis or rotting. Among all species, *F*. *pseudograminearum* and *F*. *culmorum* were the most virulent pathogens that resulted in the deaths of seedlings, which is consistent with earlier observations [13,16,20,62,71,74]. In addition to their high frequency of occurrence, isolates of *B*. *sorokiniana*, a well-known wheat pathogen, as well as *Fusarium* sp., *R*. *solani* AG2-1, and *F*. *redolens*, were considered moderately virulent. However, *B*. *sorokiniana* was more virulent than the others. Despite the fact that *R*. *solani* isolates belonging to AG2-1 were widely found in Canada [75], the United States [76], and Azerbaijan [77], they caused only mild root rot on wheat seedlings in the majority of these investigations, corroborating our findings. However, some contradictory reports also exist. For example, while AG 2-1 was consistently isolated from wheat roots in Canada, the isolates of this AG did not typically cause disease on wheat [78]. *Fusarium redolens*, on the other hand, has only been identified as a wheat pathogen in Canada [79], Turkey [80], and Kazakhstan [34].

Among the other pathogen species, *C*. *spicifera*, *C*. *inaequalis*, and *N*. *oryzae* were identified as mildly virulent pathogens of wheat. Among them, *C*. *spicifera* and *C*. *inaequalis* were also isolated as being related to crown and root rot, though to a lesser extent, which was consistent with past findings [23,65,68,81]. *Nigrospora* spp. were previously isolated with a low frequency from discolored sub-crown internodes of wheat in Canada and Azerbaijan [23,67] and *N*. *oryzae* from seed mycoflora in Kazakhstan [36]. These species, isolated from the wheat crown and root tissues, were discovered to be pathogenic to wheat seedlings for the first time. Regardless of their level of virulence, these species have the potential to convert into strong pathogenic species due to pathogenic differentiation; hence, future trends of these pathogen species must be closely monitored. Based on disease severity scores that were not statistically significantly different (*p* < 0.05) from control plants, seven *Fusarium* species, *F*. *acuminatum*, *F*. *oxysporum*, *F*. *flocciferum*, *F*. *torulosum*, *F*. cf. *incarnatum*, *and F*. *equiseti*, and *M*. *phaseolina*, were considered to be non-pathogens, which was consistent with prior findings from wheat pathogenicity studies [19,23,60,61]. However, several investigations have found isolates of *F*. *acuminatum*, *F*. *equiseti*, and *F*. *oxysporum* demonstrating the pathogenic response to wheat at varying levels, contradicting our findings [68,82].

The study is the first comprehensive assessment of fungal species associated with wheat crown and root rot carried out in Kazakhstan’s major wheat-growing regions. A variety of fungal species were assessed in this study by morphological and molecular methods, including DNA sequencing of ITS, EF-1α, and GAPDH loci. In all surveyed regions, *B. sorokiniana* was the most commonly isolated species; however, it was moderately virulent. *F*. *acuminatum*, the second in that order, was non-pathogenic. Despite their much lower frequency and field incidence, *F*. *pseudograminearum* and *F*. *culmorum* were the most and equally virulent pathogens. Among the various pathogens, *Fusarium* sp., *R*. *solani* AG2-1, and *F*. *redolens* were moderately virulent, whereas *C*. *spicifera*, *C*. *inaequalis*, and *N*. *oryzae* were mildly virulent. In addition, the study showed that in the central part of the country (Karagandy region), all *Cochliobolus* anamorphs and *F*. *acuminatum* had a higher field incidence and isolation frequency, whereas in the eastern (East Kazakhstan region) and southeastern (Almaty region) parts, all other species had a higher ratio. A comprehensive study of the fungal populations associated with this condition has never been conducted in Kazakhstan. Consequently, we could not compare our findings to previous research. The findings of this research will be useful for the development of management guidelines for root and crown rot fungi in wheat across Kazakhstan’s different agronomic zones.

## Figures and Tables

**Table 1:** The number of isolates associated with wheat crown/root rot and isolation frequency obtained from three different wheat-growing regions in Kazakhstan.

Species	Karagandy		East Kazakhstan		Almaty		Total	
	N	IF	N	IF	N	IF	N	IF
*Bipolaris sorokiniana*	222	43.61	136	43.31	189	47.49	547 *	44.80
*Fusarium acuminatum*	158	31.04	39	12.42	52	13.07	249	20.39
*Fusarium equiseti*	26	5.11	48	15.29	50	12.56	124	10.16
*Curvularia spicifera*	62	12.18	17	5.41	14	3.52	93	7.62
*Fusarium culmorum*	14	2.75	22	7.01	22	5.53	58	4.75
*Fusarium oxysporum*	5	0.98	21	6.69	24	6.03	50	4.10
*Fusarium redolens*	4	0.79	19	6.05	6	1.51	29	2.38
*Rhizoctonia solani* AG2-1	1	0.20	4	1.27	8	2.01	13	1.06
*Nigrospora oryzae*	3	0.59	3	0.96	6	1.51	12	0.98
*Curvularia inaequalis*	6	1.18	2	0.64	3	0.75	11	0.90
*Fusarium pseudograminearum*	2	0.39	2	0.64	5	1.26	9	0.74
*Fusarium flocciferum*	0	0.00	0	0.00	9	2.26	9	0.74
*Macrophomina phaseolina*	0	0.00	0	0.00	8	2.01	8	0.66
*Fusarium* cf. *incarnatum*	4	0.79	0	0.00	0	0.00	4	0.33
*Fusarium* sp.	1	0.20	0	0.00	2	0.50	3	0.25
*Fusarium torulosum*	1	0.20	1	0.32	0	0.00	2	0.16
**Total**	**509**	**100**	**314**	**100**	**398**	**100**	**1221**	**100**

Abbreviations stand for: N = Number of isolates; IF = Isolation frequency; * = The species row is ordered from the highest to the lowest based on the total number of isolates obtained.

**Table 2:** The distribution of fungal species associated with wheat crown/root rot and field incidence of fungal species across three different wheat-growing regions in Kazakhstan.

Species	Karagandy		East Kazakhstan		Almaty		Total	
	N	FI	N	FI	N	FI	N	FI
*Bipolaris sorokiniana*	25 *	96.15	13	72.22	18	85.71	56	86.15
*Fusarium acuminatum*	24	92.31	7	38.89	12	57.14	43	66.15
*Fusarium oxysporum*	5	19.23	10	55.56	12	57.14	27	41.54
*Fusarium equiseti*	7	26.92	8	44.44	8	38.10	23	35.38
*Curvularia spicifera*	11	42.31	4	22.22	3	14.29	18	27.69
*Fusarium culmorum*	4	15.38	4	22.22	4	19.05	12	18.46
*Rhizoctonia solani* AG2-1	1	3.85	3	16.67	4	19.05	8	12.31
*Fusarium redolens*	2	7.69	2	11.11	2	9.52	6	9.23
*Nigrospora oryzae*	2	7.69	2	11.11	2	9.52	6	9.23
*Fusarium pseudograminearum*	1	3.85	1	5.56	3	14.29	5	7.69
*Curvularia inaequalis*	3	11.54	1	5.56	1	4.76	5	7.69
*Fusarium flocciferum*	0	0.00	0	0.00	3	14.29	3	4.62
*Fusarium torulosum*	1	3.85	1	5.56	0	0.00	2	3.08
*Fusarium* sp.	1	3.85	0	0.00	1	4.76	2	3.08
*Fusarium* cf. *incarnatum*	2	7.69	0	0.00	0	0.00	2	3.08
*Macrophomina phaseolina*	0	0.00	0	0.00	2	9.52	2	3.08

Abbreviations stand for: N = Number of fields where individual species were identified; FI = Field incidence; * = The species row is arranged in descending order based on the total number of fields in which individual species were isolated.

**Table 3:** The pathogenicity of the fungal species identified in the present study.

Species	Number of Isolates	Average Severity Index *	Average Disease Severity (%) ***	Virulence Category ****
*Fusarium pseudograminearum*	5	3.55 ± 0.60a **	71.09	HV
*Fusarium culmorum*	5	3.42 ± 0.64a	68.37	HV
*Bipolaris sorokiniana*	5	2.83 ± 0.50b	56.60	MV
*Fusarium* sp.	3	2.25 ± 0.61bc	44.98	MV
*Rhizoctonia solani* AG2-1	3	2.21 ± 0.54c	44.12	MV
*Fusarium redolens*	4	2.20 ± 0.41c	44.05	MV
*Curvularia spicifera*	5	1.94 ± 0.56cd	38.87	MiV
*Curvularia ineaqualis*	5	1.60 ± 0.47de	32.04	MiV
*Nigrospora oryzae*	5	1.56 ± 0.50de	31.24	MiV
*Fusarium acuminatum*	5	1.37 ± 0.48e	–	NP
*Fusarium oxysporum*	5	1.36 ± 0.52e	–	NP
*Fusarium flocciferum*	5	1.29 ± 0.46e	–	NP
Control	5	1.28 ± 0.41e	–	NP
*Macrophomina phaseolina*	5	1.26 ± 0.43e	–	NP
*Fusarium torulosum*	2	1.19 ± 0.29e	–	NP
*Fusarium* cf. *incarnatum*	2	1.19 ± 0.29e	–	NP
*Fusarium equiseti*	5	1.18 ± 0.37e	–	NP

The table rows are ordered from the highest to the lowest based on disease severity scores. * Standard deviation from the mean of the values belonging to all isolates; ** Values followed by the same letter are significantly different among isolates based on Tukey’s HSD at *p* = 0.05; *** Disease severity was not calculated for the species whose mean disease average score values were in the same group as the control group; **** HV: highly virulent, MV: moderately virulent, MiV: mildly virulent, NP: non-pathogenic.

## Data Availability

All relevant data generated or analyzed during this study are included in this manuscript.
